# Trans Fat Consumption and Aggression

**DOI:** 10.1371/journal.pone.0032175

**Published:** 2012-03-05

**Authors:** Beatrice A. Golomb, Marcella A. Evans, Halbert L. White, Joel E. Dimsdale

**Affiliations:** 1 Department of Medicine, University of California San Diego, San Diego, California, United States of America; 2 Department of Family and Preventive Medicine, University of California San Diego, San Diego, California, United States of America; 3 Department of Economics, University of California San Diego, California, United States of America; 4 Department of Psychiatry, University of California San Diego, California, United States of America; University of Regensburg, Germany

## Abstract

**Background:**

Dietary trans fatty acids (dTFA) are primarily synthetic compounds that have been introduced only recently; little is known about their behavioral effects. dTFA inhibit production of omega-3 fatty acids, which experimentally have been shown to reduce aggression. Potential behavioral effects of dTFA merit investigation. We sought to determine whether dTFA are associated with aggression/irritability.

**Methodolgy/Prinicpal Findings:**

We capitalized on baseline dietary and behavioral assessments in an existing clinical trial to analyze the relationship of dTFA to aggression. Of 1,018 broadly sampled baseline subjects, the 945 adult men and women who brought a completed dietary survey to their baseline visit are the target of this analysis. Subjects (seen 1999–2004) were not on lipid medications, and were without LDL-cholesterol extremes, diabetes, HIV, cancer or heart disease. Outcomes assessed adverse *behaviors* with impact on others: Overt Aggression Scale Modified-aggression subscale (primary behavioral endpoint); Life History of Aggression; Conflict Tactics Scale; and self-rated impatience and irritability. The association of dTFA to aggression was analyzed via regression and ordinal logit, unadjusted and adjusted for potential confounders (sex, age, education, alcohol, and smoking). Additional analyses stratified on sex, age, and ethnicity, and examined the prospective association. Greater dTFA were strongly significantly associated with greater aggression, with dTFA more consistently predictive than other assessed aggression predictors. The relationship was upheld with adjustment for confounders, was preserved across sex, age, and ethnicity strata, and held cross-sectionally and prospectively.

**Conclusions/Significance:**

This study provides the first evidence linking dTFA with behavioral irritability and aggression. While confounding is always a concern in observational studies, factors including strength and consistency of association, biological gradient, temporality, and biological plausibility add weight to the prospect of a causal connection. Our results may have relevance to public policy determinations regarding dietary trans fats.

Clinicaltrials.gov # NCT00330980

## Introduction

Dietary trans fatty acids (dTFA) are primarily products of hydrogenation, a chemical process that makes (unsaturated) oils solid at room temperature [1]. They are present at high levels in margarines, shortenings, and prepared foods [2]–[4]. Adverse health effects of dTFA have been identified on lipids, metabolic function, insulin resistance, oxidation, inflammation, and cardiac and general health [5]–[20]. Advantageous associations of another class of fatty acids, long chain omega-3 fatty acids (n3FA), to behavioral outcomes have been previously reported [21]–[23].

Due to the range of their deleterious biological effects, including inhibition by dTFA of n3FA production (by inhibition of delta-6 desaturase activity) [24], [25], we theorized that dTFA may be associated with greater aggression and irritability.

## Methods

### Subjects

Of 1018 male and female adults, minimum age 20 years, screened for participation in a clinical trial of lipid-lowering therapy in a primary prevention setting (the UCSD Statin Study) [26], 945 had completed a dietary assessment prior to a baseline visit and were the target of the present assessment. Subjects were broadly sampled, however persons on lipid medications, or with very low or high LDL (<115 mg/dL or >190 mg/dL), known diabetes, cardiovascular disease, HIV, or cancer were excluded [26].

The study protocol was approved by the University of California, San Diego Human Research Protections Program. All subjects gave written informed consent.

### Dietary Trans Fatty Acid Estimation

Nutrient data were collected using a food frequency questionnaire developed by the Nutrition Assessment Shared Resource of the Fred Hutchinson Cancer Research Center [27]. Consumption frequency and portion size are queried, for a series of food categories, each in turn defined by a series of foods or beverages. Additional questions relating to food preparation and purchasing further refine nutrient calculations (http://www.fhcrc.org/science/shared_resources/nutrition/ffq/).

Nutrient calculations were performed using the Nutrient Data System for Research software version 4.03, developed by the Nutrition Coordinating Center, University of Minnesota Food and Nutrient Database (version 31, released November 2000), which added *trans* fatty acid values in 1998. “T*rans*-fatty acid values were determined for all foods in the database (0% missing) and include individual contributions of 16∶1 *trans* (*trans*-hexadecenoic acid); 18∶1 *trans* (*trans*-octadecenoic acid); and 18∶2 *trans* (*trans*-octadecadienoic acid), which encompasses *cis-trans*, *trans-cis*, and *trans-trans* forms; as well as total *trans*-fatty acids. The USDA table “Fat and Fatty Acid Content of Selected Foods Containing *Trans*-Fatty Acids”… was the primary source of *trans*-fatty acid information for assignment of values to foods in the database. Additional data sources included other nutrient databases and articles in the scientific literature containing *trans*-fatty acid values for US foods, using appropriate methodologies” [28].

The study period (1999–2004) was advantageous because dTFA values were available in the nutrient database, while trans fat composition in foods was relatively stable (USFDA trans fat labeling requirements were implemented later, Jan 1 2006 [29]).

### Behavioral Endpoints

The following validated instruments were used:

- **Overt Aggression Scale Modified – Aggression subscale (OASMa)** [30]–[34]. the primary designated aggression measure, it inquires about actual aggressive behavioral actions in the prior week.
- **Life History of Aggression (LHA)** [35]. examines behavioral aggression over the subject's lifetime, generally excluding childhood violence.
- **Conflict Tactics Scale (CTS)** [36]–[38]. examines tactics, including aggressive ones, employed by (or against) the subject (behavioral hostility or hostile attribution) in the prior 2 weeks. We employed the portion eliciting behaviors by the subject.
- **Impatience**. measures self-report of subjective impatience, asking subjects to rate their impatience on a scale of 0 (not present) to 10 (maximally present).
- **Irritability.** measures self-report of subjective irritability, asking subjects to rate their irritability on a scale of 0 (not present) to 10 (maximally present).

The latter two instruments have been validated in this study (construct, convergent, predictive validity; information available on request).

### Covariates

Potential confounders of relevance to aggression assessments (and related behaviors) included age and sex [39], education, alcohol [39], [40], and smoking [40]. Exercise was examined by two measures: self-rated activity relative to others your age (5 point Likert scale from much less active through about the same to much more active – in models that also adjust for age); and the number of times in a week exercised vigorously for at least 20 minutes.

### Analyses

The association of dTFA to aggression was assessed cross-sectionally at baseline, prior to any study-related treatment. An additional prospective analysis capitalized on subsequent assessments of aggression in the placebo group. Regression analyses were performed for each behavior/aggression outcome, unadjusted and adjusted. The “full” model for each (using heteroskedasticity-independent robust standard errors, aka “White” standard errors [41]) adjusted for reported aggression predictors (age, sex, education, alcohol, smoking) and dTFA, allowing for contrast of consistency of impact of different aggression predictors, including dTFA. Both least squares regression and ordinal logit were performed for each outcome, assessing the robustness to regression approach (including for outcomes where either regression approach could be justified and, purely for illustration of robustness, those for which one specification was conceptually preferred). Exercise variables were evaluated as potential confounders.

Additional analyses examined impact of stratification by sex, ethnicity and age on the relationship between dTFA and OASMa (the primary aggression endpoint).

Prospective prediction was also assessed, restricted to the placebo group for simplicity of interpretation (averting concerns that the intervention could modify the results – though results of analysis including the total sample are also provided). dTFA at baseline were examined relative to OASMa at follow-up (6 months after baseline), adjusting for baseline OASMa.

A prior study looked at trans and saturated fats on depression; we added this combined analysis (and also examined depression). Trans fat consumption relates strongly to linoleic acid consumption; we evaluated the effect of each after removing the contribution by the other; and also examined a model adjusting simultaneously for the joint trans-saturated variable and the linoleic variable.

## Results

Baseline characteristics are shown in **Table 1**.

**Table 1:** Baseline Subject Characteristics (N = 945)*.

	Sample Mean (SD) or %
Age (years)	57.2 (12.0)
Male	68.3%
Caucasian	80.1%
Education (scaled 1–9)†	5.82 (1.50)
Current Smoker	7.94%
Alcohol (g/day)	9.64 (14.9)
Trans fat Consumption (g/day)	3.49 (2.47)

* Subjects with trans fat measurement, out of 1,016 subjects appearing for baseline participation in a clinical trial.

† Education was scored on a 9-point scale, with 1 = grade school or less, 9 = doctoral degree.

Strongly significant interrelations among the behavioral endpoints (p<0.001) but modest correlation coefficients affirm that the aggression measures we employed tapped related but distinct constructs (**Table S1 and S2**).

Regression analysis showed a strong association between dTFA and each aggression-related outcome. This association was retained after adjustment for other predictors, and was more consistently predictive other assessed aggression predictors (**Table 2** and **Table 3**). Coefficients were similar for men and women (except for irritability) though significance was greater for men, who were twice as numerous in the sample. Significance was upheld across age and ethnicity strata. Regression coefficients for dTFA as a predictor of OASMa by age were: β = 0.53 p = 0.003 (age 20–40); β = 0.27 p = 0.003 (age 40–60); β = 0.27 p = 0.008 (age>60); by ethnicity: β = 0.30 p<0.001 (Caucasian); β = 0.31 p = 0.037 (non-Caucasian).

**Table 2:** Dietary Trans Fat is Significantly Linked to Aggression Measures.

Aggression Measure		Dietary Trans FatsRelation to That Measure*		
Variable	Mean Value ± SD	Dietary Trans Fats (grams/day)		P-value
		β	SE	
**OASMa**	2.50**±**4.72	0.296	0.092	0.001
**Conflict Tactics Scale**	1.08**±**1.70	0.101	0.031	0.001
**Life History of Aggression**	10.1**±**6.93	0.277	0.106	0.009
**Impatience**	1.87**±**2.12	0.0953	0.0372	0.011
**Irritability**	1.30**±**1.83	0.084	0.031	0.007

OASMa: Overt Aggression Scale Modified – aggression subscale.

* In regression with robust standard errors, adjusted for age, sex, education, alcohol and smoking.

**Table 3:** Significance of Dietary Trans Fats Along with Other Potential Aggression Predictors in Multivariable Adjustment†.

Predictor	Aggression Measures				
	OASMa	LHA	CTS	Impatience	Irritability
**Age**	**/**	**/**	*/−	*/*	**/**
**Male**	−/−	**/**	−/−	−/−	−/−
**Education**	*/**	+/*	−/−	−/−	−/*
**Smoking**	−/−	−/−	−/−	*/+	+/−
**Alcohol**	−/−	**/**	−/−	**/*	*/*
***Trans Fats***	* */* **	** */* *	** */* **	* */* *	** */* **

OASMa: Overt Aggression Scale Modified – aggression subscale.

LHA: Life History of Aggression. CTS: Conflict Tactics Scale.

− No significant or borderline association.

+ 0.05<p<0.1 (borderline significant).

* 0.01<p<0.05 (significant).

** p<0.01 (highly significant).

† Significance levels shown are results (respectively) of linear regression/ordinal logit analyses (both with robust standard errors [41]). Analyses for each aggression outcome are adjusted for each predictor variable.

dTFA significantly predicted future aggression, in adjusted prospective analysis confined to the placebo group. This was true even after also adjusting for aggression at time of dTFA assessment (factoring out the association of baseline aggression with dTFA): β = 0.15 p = 0.041. This contrasted with other predictors, for which the impact on aggression at baseline largely accounted for the impact at follow-up. (The central follow-up analysis presented here was in the untreated placebo group. However dTFA also prospectively preedicted aggression in the statin group: β = 0.25, p = 0.032; and in the total sample, adjusted for active treatment, as well as baseline and on-treatment cholesterol: β = 0.21 p = 0.009.) Regarding other potential lifestyle confounders, exercise variables (×2) had no relationship to aggression variables (×5) with one exception: vigorous activity for 20 minutes predicted CTS (one of 10 tested pairings), but the trans fat relationship retained its potent prediction of aggression with additional adjustment for this (p = 0.001). Linoleic acid (pfa18∶2) was collinear with trans fats, but the residual transfat contribution, after separating out the shared contribution with pfa18∶2 remained strong, 0.23, and significant, p = 0.002. Others have considered trans and saturated fat variables together for mood associations. A fused trans-saturated fat variable bore a smaller coefficient consistent with the lower saturated fat coefficient; significance remained strong (p = 0.001). The pfa18∶2 coefficient lost significance (and the coefficient was negative), when both the trans-saturated variable and the pfa18∶2 variable were included in the model.

## Discussion

dTFA showed a significant association to behaviors that have unfavorable repercussions to others – indeed dTFA were more consistently predictive than that of other assessed and recognized aggression predictors. The effect was robust to adjustment for potential confounders like age, education, smoking, and alcohol. This is the first study to show a connection of dTFA to aggression.

A role for nutrition and specifically fatty acids in behavior [42] has been previously reported [43]. Of note, dTFA variably obstruct production of docosahexaenoic acid, a long chain n3FA that has protected against aggression-related outcomes in some observational and experimental studies [21]–[23]. In one animal study the effect of dTFA on n3FA themselves did not extend to the brain [44]. However, it is unknown whether n3FA behavioral benefits reflect purely direct effects of n3FA on the brain or indirect effects – e.g. through effects on prostaglandins and oxidative and inflammatory mediators that may themselves cross the blood-brain-barrier [45]–[47]. Indeed, dTFA mechanisms that could have implications for aggression include cell energy alterations [18], oxidative stress [15] and inflammatory effects [10], [11]. n3FA have also been linked to lower depression risk, and analogous reasoning might yield the hypothesis that trans fats may adversely affect depression. Indeed, a relation of dTFA and saturated fats to depression has been reported [48]. The association to depression was evident in our sample as well (based on the Center for Epidemiologic Studies Depression Scale); but our focus is on the aggression association, which, in our sample, is considerably stronger. Of note, n3FA intake was not significantly related to aggression in our sample.

As with all observational studies, there are limitations including the potential for unmeasured confounding. However, observational data will likely be central to exploring this issue as human intervention studies with randomized dietary trans fat allocation are unlikely to be pursued due to ethical concerns, given evidence of other adverse health effects of dTFA.

This study did not employ objective markers of trans fatty acids such as red blood cell membrane trans fats [49], or plasma phospholipid trans fats [50], and future studies should seek to employ these measures to establish whether the relationship is upheld. Use of these as biomarkers of dTFA [51], may correlate imperfectly to dTFA. However, while serum markers are “objective,” they also have limitations relative to dTFA. (Other factors may affect serum level and outcomes, creating an apparent connection that need not have anything to do with actions of the nutrient. For illustration, low serum levels of iron may be linked to colon cancer death, or death from exsanguination, but it is neither low iron intake nor low iron levels that *produce* these. Rather, colon cancer may cause GI bleed and low measured iron *at a given iron intake level*, producing the appearance of a connection.) Since one can intervene upon diet, the association of specifically dTFA to behavior remains of independent importance.

Trans fat consumption was estimated from dietary recall. Not all foods that go by the same label have the same trans fat content. However, provided the misclassification is nondifferential, this would be expected to produce bias toward the null and could not explain strong and significant findings. Moreover, this study was done during a period of relative stability of trans fat content in foods (1999–2004) prior to more recent efforts to restrict dTFA – a comparative strength of this investigation.

Trans fat consumption could serve as a marker for other untoward behaviors and practices that could in turn be linked to aggression. This concern is somewhat mitigated as our finding emerged in a clinical trial sample and was preserved with adjustment for other adverse health behaviors such as smoking and alcohol. Triangulating evidence and other studies will be required to more confidently establish whether the association is causal.

This work also has important strengths. The premise (and results) are original but rest on a biological foundation. Factors including the strength of association, consistency, temporality (prospective prediction), biological gradient (“dose response”), biological plausibility, and coherence with other literature – factors such as those in Hill's presumptive criteria for causality with observational data – were evident in our findings, and add weight to the possibility that the association we identify could have a causal basis; but confounding cannot be excluded. If the association is causal, the findings provide one further potential explanation for the recognized association between hostile/aggressive behaviors and heart disease. Trans fats could serve as common cause for both outcomes [20], [52]–[54] (analogous to the observation that n3FA may serve as a common protection against both [43]).

If the association is determined to be causal, then the detrimental effects of trans fats may extend beyond the person who consumes them to affect others with whom that person interacts. Should that prove to be the case, the inclusion of synthetic trans fats in diets may bear reexamination – with implications to public policy and regulation.

## Supporting Information

Table S1 OASMa: Overt Aggression Scale Modified – aggression subscale. LHA: Life History of Aggression. CTS: Conflict Tactics Scale. * P<0.0001 for all correlations.
(DOC)

Table S2 OASMa: Overt Aggression Scale Modified - aggression subscale. LHA: Life History of Aggression. CTS: Conflict Tactics Scale. Power was greater for men, who represented 68% of the sample (about twice as many men as women). * Significant change in aggression shown in bold (P<0.05).
(DOC)
