# Supplementary material for: Trans Fat Consumption and Aggression
Source: PLoS One. 2012 Mar 5;7(3):e32175. doi: 10.1371/journal.pone.0032175 (PMC3293881; doi:10.1371/journal.pone.0032175)
Supplement: Table S1 — OASMa: Overt Aggression Scale Modified – aggression subscale. LHA: Life History of Aggression. CTS: Conflict Tactics Scale. * P<0.0001 for all correlations. (DOC) [file pone.0032175.s001.doc]

**Table S1. Correlations Among Aggression Measures***

**(baseline)**

|  | **OASMa** | **LHA** | **CTS** | **Impatience** | **Irritability** |
| --- | --- | --- | --- | --- | --- |
| **OASMa** | 1 |  |  |  |  |
| **LHA** | 0.188 | 1 |  |  |  |
| **CTS** | 0.451 | 0.338 | 1 |  |  |
| **Impatience** | 0.286 | 0.274 | 0.323 | 1 |  |
| **Irritability** | 0.367 | 0.253 | 0.276 | 0.692 | 1 |
