# Supplementary material for: Trans Fat Consumption and Aggression
Source: PLoS One. 2012 Mar 5;7(3):e32175. doi: 10.1371/journal.pone.0032175 (PMC3293881; doi:10.1371/journal.pone.0032175)
Supplement: Table S2 — OASMa: Overt Aggression Scale Modified - aggression subscale. LHA: Life History of Aggression. CTS: Conflict Tactics Scale. Power was greater for men, who represented 68% of the sample (about twice as many men as women). * Significant change in aggression shown in bold (P<0.05). (DOC) [file pone.0032175.s002.doc]

**Table S2. Dietary Trans Fat Relation to Aggression Measures, by Sex (Age Adjusted)**

| **Aggression Measure** | **Female** | | | **Male** | | |
| --- | --- | --- | --- | --- | --- | --- |
|  | **β** | **SE** | **P** | **β** | **SE** | **P** |
| **OASMa** | 0.270 | 0.173 | 0.119 | 0.300 | 0.065 | **<0.001*** |
| **LHA** | 0.403 | 0.189 | **0.034*** | 0.329 | 0.104 | **0.002*** |
| **CTS** | 0.170 | 0.064 | **0.009*** | 0.110 | 0.040 | **0.006*** |
| **Irritability** | 0.00655 | 0.0598 | 0.913 | 0.095 | 0.027 | **0.001*** |
| **Impatience** | 0.085 | 0.075 | 0.259 | 0.107 | 0.032 | **0.001*** |
